# Exploration of Estimated Emigration Trends of Polish Health Professionals

**DOI:** 10.3390/ijerph19020940

**Published:** 2022-01-14

**Authors:** Alicja Domagała, Marcin Kautsch, Aleksandra Kulbat, Kamila Parzonka

**Affiliations:** 1 Institute of Public Health, Faculty of Health Sciences, Jagiellonian University Medical College, 31-008 Krakow, Poland; marcin.kautsch@uj.edu.pl (M.K.); kamila.parzonka@student.uj.edu.pl (K.P.); 2 Faculty of Medicine, Jagiellonian University Medical College, 31-088 Krakow, Poland; alexandra.kulbat@gmail.com

**Keywords:** doctors, nurses, physiotherapists, pharmacists, health professionals, healthcare system, emigration, emigration intentions

## Abstract

Background: Due to the significant staff shortages, emigration of health professionals is one of the key challenges for many healthcare systems. Objective: The aim of this article is to explore the estimated trends and directions of emigration among Polish health professionals. Methods: The emigration phenomenon of Polish health professionals is still under-researched and the number of studies in this field is limited. Thus, the authors have triangulated data using two methods: a data analysis of five national registers maintained by chambers of professionals (doctors, nurses, midwives, physiotherapists, pharmacists, and laboratory diagnosticians), and data analysis from the Regulated Profession Database in The EU Single Market. Results: According to the data from national registers, between 7–9% of practicing doctors and nurses have applied for certificates, which confirm their right to practice their profession in other European countries (most often the United Kingdom, Germany, Sweden, Spain, and Ireland). The relatively high number of such certificates applied for by physiotherapists is also worrying. Emigration among pharmacists and laboratory diagnosticians is rather marginal. Conclusions: Urgent implementation of an effective mechanism for monitoring emigration trends is necessary. Furthermore, it is not possible to retain qualified professionals without systemic improvement of working conditions within the Polish healthcare system.

## 1. Introduction

In May 2004, Poland joined the European Union, which significantly changed the labour market for health professionals. The free movement of people (based on Directive 2005/36/EC) meant that professional qualifications obtained at Polish universities were recognized throughout the EU, which reduced barriers related to emigration [1]. Since 2004, doctors, nurses, midwives, dentists, and pharmacists trained in Poland and holding Polish nationality have been free to seek employment, work, and settle down in any European Union Member State. Free mobility of medical staff is legally binding and may not be hindered by governments or other health sector stakeholders [2].

Migration is a very common phenomenon. According to the latest available Eurostat data, in 2019, 4.2 million people immigrated to one of the Member States, and 2.7 million emigrated from one of the above-mentioned countries. Some 5.1% of the 447.3 million people living in the EU on 1 January 2020 were non-EU citizens [3].

Pull and push factors can stimulate migration. The bigger the differences between a country of origin and a destination country, the stronger that stimulation is. Migrants have to, therefore, assess their current professional and economic circumstances (for instance, demotivating working conditions and/or low salaries) and hoped for change and the opportunity for professional development. They have to weigh career opportunities and expected prosperity against the costs of leaving their country, which means both psychological and social costs [4].

Migration (including emigration and immigration) also applies to healthcare professionals. Something that distinguishes the migration of healthcare personnel from the migration of other professional groups is the need for migrants to present relevant qualifications, which must be documented if a person wants to work in their profession. In the case of emigrants from European Union Member States, this is facilitated by the fact that the professional qualifications obtained by these people are recognized by all Member States due to the above-mentioned EC Directive [1].

Migration issues can and should be analysed from a number of points of view. This applies, for example, to issues such as the health of migrants [5], the processes of their adaptation and acculturation [6], the reaction of the local population, the possible increase in phenomena such as xenophobia [7] and, consequently, the growing popularity of nationalist parties [8]. Different anti-immigration parties present common discursive patterns, particularly in their framing of the immigrants as “others” [9]. Researchers in political discourse analysis emphasise the need for further investigations on inequity, the well-being of emigrants, and the safety and security of their living and working conditions [9,10]. The importance of that phenomenon and its implications for the whole economy requires governmental responses. As the research shows, migration policies of different countries can differ substantially, depending on local circumstances [11].

Due to the significant shortage of health care personnel in Poland, this article focuses on the quantitative issues describing this phenomenon of emigration of health care personnel from this country in relation to the main professional groups and the estimated trends that have accompanied them in the last dozen or so years. By analysing the origins of emigration in Poland, one can state that they are quite typical for countries of similar or lower economic status [12]. Low salaries seem to be quite an obvious reason to emigrate. For the same reason, Poland does not attract foreign health professionals, which is untypical for other Organisation for Economic Cooperation and Development (OECD) countries. In 2016, the proportion of foreign-born health professionals across 18 OECD countries for which data are available reached the level of 27% for doctors and 16% for nurses. The same indicators for Poland were respectively 2.7% and 0.2% in 2011 (no data for later years is available). Some countries—for instance Switzerland, Ireland, or the United Kingdom—to name just some of the European countries, are heavily dependent on foreign-born doctors and nurses [13]. At the same time, the relatively low cost of living and studying in Poland and the restrictive quota of persons allowed to study medicine in other countries (for instance, Norway) attracts medical students from these countries to study in Poland (see Section 3.2). A similar phenomenon can be observed in Hungary and Romania [13].

For many years, one of the main destination countries for Polish emigrants was the United Kingdom [14]. What is (and will) be the impact of Brexit on health professionals’ migration? Admittedly, this question is not the subject of this article’s analysis, but it could have an impact on the migration trends of Polish health professionals. Based on data from the Annual Population Survey it has been estimated that in 2018, there were about 1.9 million professionals employed in the healthcare sector in the UK, including 88% British nationals, 6% EU nationals, and 6% non-EU nationals [15]. Among them, physicians is the professional group with the highest proportion of non-British nationals. According to the NHS data, approximately 29% of physicians and 18% of nurses were non-British nationals. The NHS needs the essential contribution of migrant health professionals: there are currently over 100,000 vacancies for nurses and several thousand for physicians [15].

The comparatively low salaries of medical personnel and poor working conditions has made Poland a source, not a destination country for medical staff. The relatively low expenditure on healthcare in Poland, compared to other EU Member States, also causes significant social tensions. Expenditure on health in Poland in 2019 was 6.2% of GDP, compared to 8.8% in the OECD European region average [16]. Various types of conflicts between health professionals and the government, local governments, or individual healthcare facilities have been ongoing practically throughout the last three decades. Salaries of medical specialists with long experience are at a relatively high level; however, they are usually employed in several places (average 1.9) and work long hours (average 165 h per month and 234 h per month including additional shift work) [17], which is 22% more than stipulated by the European Working Time Directive (EWTD) [18]. Other groups of health professionals, including junior doctors, nurses, physiotherapists, and paramedics still feel that they are significantly underpaid. The average remuneration of a specialist in 2017 was 1.4 times the average salary in Poland, which was the lowest among OECD countries [19]. The low level of average remuneration of Polish health professionals compared to other OECD countries is also confirmed by indicators published in International Health Data Comparison [20].

The discussed situation and growing expectations regarding salaries has resulted in relatively frequent strikes, or strike readiness, to force the government to raise wages. Demands for salary increases and their introduction by the government have not been agreed to by the management of healthcare facilities and there have been no dedicated funds for such increases. It can be concluded that various types of solutions are being implemented “above the heads” of the people responsible for these institutions—their directors and the local government units that own the majority of public healthcare facilities, not to mention the public payer. According to the logic of the Polish insurance system, healthcare facilities contract their services with the National Health Fund. The public payer finances the above-mentioned contracts using funds generated by collecting premiums. These premiums are based on a percentage of the salary/income of all insured people, currently 9%, and have not changed in years. For this reason, healthcare professional raises could not be introduced or have been introduced piecemeal in several phases, causing further dissatisfaction of the above mentioned professional groups.

Physicians, nurses, and other frontline health professionals in each country have been the heroes of the COVID-19 pandemic and the significance of healthcare systems and medical staff to the survival of society has never been clearer [21]. On the other hand, the COVID-19 pandemic has shown that substantial staff shortages and the resulting excessive workload and fatigue of health professionals constitute a real threat to the delivery of essential health services and the effective fight against the pandemic.

The COVID-19 pandemic had a strong impact on the health workforce, including workload and fatigue; long-term psychological stress in the working environment, often leading to burnout; increased risk of infection (especially before vaccinations were available), and the need to update knowledge rapidly and constantly about the new virus. Moreover, discrimination, stigmatization, and violence against health workers related to COVID-19 have been reported [22]. Growing problems in the healthcare sector and the related excessive workload of medical staff have led to employee fatigue and decreased job satisfaction. In this situation, and taking into account the problems that especially the youngest health professionals experience in Poland, an increase of emigration intentions could be expected among members of this group. Therefore, decisive, urgent actions are needed in this regard. To protect and support the health workforce in this extremely difficult situation and prevent long-term consequences on their physical and mental health, urgent actions, real support, and capacity-building are needed [23,24,25,26]. According to WHO recommendations, the interventions should include protecting and supporting health professionals, building capacity, redefining professional roles, ensuring real support in the work environment and a manageable workload, improving availability, and rationalizing distribution, strengthening workforce policy, and adjusting legal regulations [27].

The current situation of Polish medical staff is characterised by a low rate of employment, ageing, low professional prestige, and low remuneration. The main challenges are listed below:

- One of the lowest employment rates of medical professions per capita in Europe: 2.4 doctors/1000 inhabitants (EU average: 3.8/1000) and 5.1 nurses/1000 inhabitants (EU average: 8.2/1000). These indicators have not changed for many years, which makes the staff deficit extremely severe due to the care demand of the ageing Polish society [28].
- An increasing age of health professionals and a systematic increase in the share of people of retirement age in the cohort. This results from a lack of generational replacement in the most numerous groups of medical professions. Currently, the average age of a doctor is 50.2 (specialists, over 54.2); the average age of a midwife is 50.4, and that of a nurse is 52.6 [22]. The latter is growing the fastest of them all.
- Excessive workload and the need to take up employment in several places (over 60% of doctors and approximately 30–40% of nurses work in more than one healthcare unit) [22].
- Low prestige and an unsatisfactory level of remuneration (especially among junior doctors, nurses, paramedics, and physiotherapists, whose wages in Poland are perceived as unsatisfactory). To improve the remuneration policy, in 2017 the Act on the method for determining the lowest basic salary for employees performing medical professions employed in healthcare entities was implemented, regulating the minimum basic salaries of medical staff employed in healthcare facilities [29].
- Recognition of physiotherapists as a medical profession (the third largest group), and the establishment of a chamber representing the interests of this important professional group [30].

In 2017, Polish residents (doctors under residency training), frustrated with the heavy workload, staff shortages, poor working conditions, and barriers in their career paths, have organised different forms of protests demanding changes. Their key demands have been greater participation in decisions about the system, increasing public healthcare expenditure (from the current of 4.8% to at least 6.8% of gross domestic product, GDP), reducing administrative burdens, increasing the size of the health workforce, improving working conditions, and pay rises. Other professions, such as paramedics, nurses, midwives, and physiotherapists have also organised numerous strikes focused on improvement of working conditions, increasing salaries, and reducing bureaucracy.

The low salaries mentioned above (in relation to salaries in Western European countries), tough working conditions (long working hours, excessive workload) and limited career development prospects are believed to be the main factors contributing to the emigration of medical professionals from Poland to other countries.

The report published by the Polish Supreme Audit Office highlights that there is a lack of monitoring health professional emigration, and the real scale of this phenomenon is unknown [31]. In Poland there are no tools or mechanisms to gather statistical data and monitor flows of health professionals. Current data is based only on the number of certificates issued by the chambers of particular health professionals, which confirm persons’ qualifications for the purpose of the authorized right to practice in other EU Member States [13,31,32,33]. These are only an estimation, not a direct measurement of real health professional emigration.

In addition, the literature review has indicated a limited number of quantitative research concerning emigration intention of Polish practicing health professionals since accession to EU. These were conducted among nurses [34,35] and doctors [35,36]. According to the results reported by Szpakowski et al., 29.6% of 581 nurses participating in the study intended to emigrate from Poland for professional reasons [34]. In the next study, conducted two years later by Szpakowski et al., 16% of doctors and 15% of nurses declared the intention to emigrate from Poland in the coming years [35].

In the study focused on hospital physicians, out of 1003 respondents, 273 declared the intention to migrate (4.5% answering ‘definitely yes’, 22.7%: ‘probably yes’) [36]. Results of this study show that among specialists, only 2.7% answered the question of intention to migrate ‘definitely yes’ compared to 8.33% among doctors under residency training. Furthermore, specialists more often denied the intention to migrate compared to younger doctors, which was also confirmed in the study conducted by Szpakowski et al. [35]. The main destination countries were similar in all studies: Germany, the United Kingdom, Ireland, Norway, and Sweden. Moreover, the main reasons for emigration were similar: higher earnings abroad, better working conditions and work-life balance abroad, the ineffective organisation of the healthcare system in Poland, poor working conditions, barriers in career paths, and the lack of professional opportunities [34,35,36].

This study aims to explore the estimated trends and directions of emigration among Polish health professionals. Two research questions have been defined:

Q1: What are the estimated emigration trends of Polish health professionals?

Q2: Which European countries are the main destinations of Polish medical staff?

## 2. Materials and Methods

### 2.1. Study Design and Participants

To answer the first research question, a cross sectional study covering all medical chambers registered in Poland has been conducted. The first step focused on gathering data from all five national official registers maintained by associations of health professionals: (1) the National Chamber of Physicians, (2) the National Chamber of Nurses and Midwives, (3) the National Chamber of Physiotherapists, (4) The Supreme Pharmaceutical Chamber, and (5) the National Chamber of Laboratory Diagnosticians. Membership in a chamber is mandatory for all practicing health professionals (except paramedics, who still do not have their own chamber). National chambers maintain central registers of licensed (with the right to practice) and actively practicing professionals. In March 2021, the first author of this article asked all national chambers for official data concerning the number of certificates issued to their members since EU accession. All five national chambers responded positively to this written request and in April 2021 provided the statistical data.

In order to answer the second research question, the European database dedicated to medical staff emigration was explored [33]. We analysed the data reported in the European Commission Regulated Profession Database since May 2004 and we collected data on the number of professionals who had received their diplomas in Poland and confirmed their professional qualifications (having them formally recognized) in other countries.

Inclusion criteria were positive automatic sectoral professions, positive automatic general system, positive after aptitude test, and positive after adaptation period. Exclusion criteria were negative automatic sectoral professions, negative automatic general system and professional experience, negative after aptitude test, negative after adaptation period, appeal, undergoing adaptation period, and being examined.

### 2.2. Data Sources and Variables

Based on the EU Directive 2005/36/EC, physicians, dentists, nurses, midwives, physiotherapists, pharmacists, and laboratory diagnosticians can apply (within their regional professional chambers) for certificates confirming their professional qualifications, which grant the legal right to practice in other European countries [1]. The gathered data reported information concerning the numbers of certificates issued by the national chambers, which confirm professional qualifications in order to authorise the legal right for health workers to practice their profession in other European Union countries. Based on the data, we have analysed the trends in the number of requested certificates between 2004 and 2020 separately for each professional group.

The next source of our analysis was the database: The EU Single Market, in the Regulated Profession database [33]. This database reports information on regulated professions and the statistics on migrating professionals, presenting the number of positive decisions taken on recognition of professional qualifications for the purpose of permanent establishment, as provided by EU Member States and EEFA countries (Iceland, Liechtenstein, Norway, and Switzerland). Currently the European Commission is in the process of updating some of the content on this platform following the withdrawal of the United Kingdom from the EU. However, since the UK is an important emigration destination for Polish medical staff, it is presented separately in our analysis.

### 2.3. Statistical Analysis

The Shapiro–Wilk statistical test was performed and histograms were prepared showing the distribution of the studied variables to assess whether the data present normal growth. For variables with a normal distribution, the mean and standard deviation were calculated, while for variables whose distribution was not consistent with the normal distribution, the median and IQR were calculated. The statistical analysis was prepared using the STATISTICA 13.3 software. A statistically significant result was one whose significance level was *p* < 0.05. In addition, we have analysed the trends in the number of requested certificates between 2004 and 2020 separately for each professional group. To examine the changes associated with the number of certificates issued for all examined medical professions, a Joinpoint regression analysis was performed, allowing for significant changes in trends during the study period to be detected. The results are presented as annual percentage change (APC) with 95% confidence interval (95% CI).

## 3. Results

### 3.1. Estimated Emigration Trends of Polish Health Professionals in the Period of 2004–2020

As stated in the introduction, due to the lack of comprehensive and consistent data on emigration in Poland, this estimation is based on the number of certificates issued by the regional chambers of particular health professionals, confirming qualifications that grant the legal right to practice in other EU countries. These documents rather indicate ‘intention-to-leave’, but there is no better measurement of this phenomenon. Data obtained from five national chambers of health professionals is presented in the sections below.

**Figure 1:**
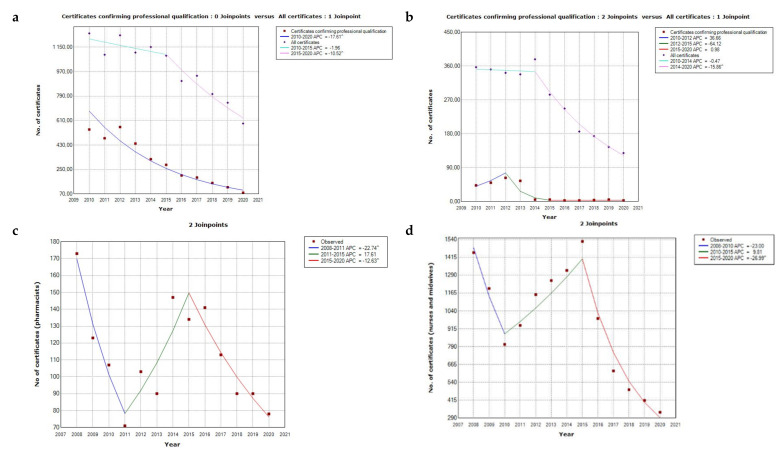
The annual percentage change in the number of certificates issued to: (**a**) doctors; (**b**) dentists; (**c**) pharmacists; (**d**) nurses and midwives. ^—significant trend (<0.05).

The trend lines (Figure 1), the data from Table 1 and Table S1 in the Supplementary Material show how the number of certificates collected by doctors, dentists, nurses, midwives, physiotherapists, and pharmacists changed between 2004 and 2020. In the years of 2004–2009, the most certificates, almost 12,000, were issued to nurses and midwives. The number of certificates issued to doctors was slightly less—7805. The scale of issued certificates among dentists was much lower; during these years they only downloaded a little over 800 certificates. Data for physiotherapists have been collected since 2017 and the number of certificates issued for physiotherapists is significant (around 200–300 a year). Despite the pandemic in 2020, this number did not decrease and 239 certificates were issued. In the case of physiotherapists, the obtained data covered a narrow period of time; they were not presented in the article either in a trendline chart or in using the annual percentage change.

Information about the number of all certificates (confirming professional qualification and certificates of good standing) issued for doctors and dentists from 2004–2020 is presented in the Supplementary Material in Table S1. Persons who had been issued at least one type of certificate—confirming their qualification or a certificate of good standing (valid for only 3 months)—were also taken into account (Figure 1a,b). Details on statistical analysis for the change in the number of certificates issued to doctors, dentists, nurses, midwives, and pharmacists are presented in Table 2.

#### 3.1.1. The National Chamber of Physicians Data

Charts with annual percentage change (Figure 1a,b) show the change in the number of certificates for the recognition of professional qualifications received by doctors and dentists. For the years 2004–2009, only aggregated data on the number of issued certificates are available; therefore, this information has not been included in the chart. This figure was 7805 for doctors and 810 for dentists. Figure 1a presents two curves that reflect the annual percentage change in the number of issued certificates. The number of certificates confirming the professional qualifications of doctors marked in blue shows one line with a constant decrease, APC = −17.605, (*p* value < 0.001). The percentage change concerning all certificates (including good standing) was illustrated with two lines. The line for the time period from 2010 to 2015 shows a slight and not significant decrease; the line for the period 2015 to 2020 has a more negative slope, APC = −10.517 (*p*-value equal 0.003).

The number of all certificates collected by doctors and dentists (including those of good standing) was characterised by a normal distribution. The average number of all certificates issued to doctors was 991.3 (SD = 214.) and 266.5 (SD = 98.3) for dentists. The distribution of the number of certificates confirming the qualifications of dentists was not consistent with the normal distribution. The median was 21.7 and the IQR was 47. In the following years, a steady decline in issued certificates can be observed. For the period from 2010 to 2014; the APC was −0.468, and the *p* value was 0.807, which corresponds to a slight decrease, which was not statistically significant; and in 2014–2020 the annual percentage change was equal to −15.857 and the *p* value was <0.001, which indicates a much larger and statistically significant decrease in the number of issued certificates than in the previous years. The annual percentage change line for dentists consists of 3 sub-points, a line with a positive slope for 2010–2012, APC = 36.664, and a *p*-value of 0.445; the line for the period from 2012 to 2015 with a clearly negative slope shows value of annual percentage change equal to −64.124 and at value of *p* almost statistically significant equal to 0.064. The last segment of the red line shows the change for 2015–2020; the APC is close to 1 (APC = 0.976), and the line showing the change in time is almost horizontal, because the number of certificates issued over the years had remained almost the same. For the years 2004–2009, the figure was 16,032 for doctors and 4263 for dentists. In the following years, a steady decline in issued certificates can be observed. Nevertheless, the number of certificates issued in 2020 was influenced by the outbreak of the pandemic and travel restrictions, so an even greater decline can be seen than before.

#### 3.1.2. The National Chamber of Nurses and Midwives Data

According to the data obtained from the National Chamber of Nurses and Midwives, between Poland’s EU accession and 2020, over 21.8 thousand nurses and midwives asked for certificates, which confirm their legal right to practice their profession in other EU countries.

**Figure 2:**
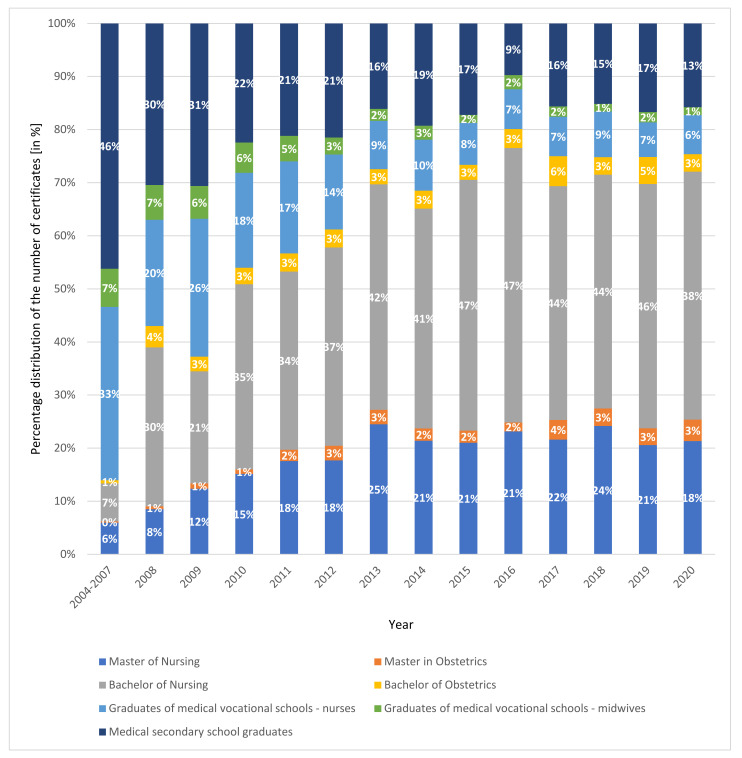
Percentage distribution of the number of certificates issued in the period of 2004–2020 for nurses and midwives. Based on data from the National Chamber of Nurses and Midwives.

The largest number of certificates was gathered in the years 2004–2007, i.e., right after Poland joined the European Union (Figure 2); the number of certificates issued at that time was 9316 which means some 2300 per year on average. In Figure 1d, three different trends are observed over time. In the period of 2008–2010, there was a downward trend, the annual percentage change in the number of issued certificates amounts to −23, and although the *p* value is above 0.05 (0.082), there was a decreasing tendency. From 2010 to 2015 there was a noticeable increase in the issued certificates, the APC = 9.812 and the *p*-value, although it indicates a statistically insignificant difference, was close to 0.05 (*p* = 0.057), and it can be concluded that there was an increasing tendency. The annual percentage change for 2015–2020 was a statistically significant change, APC was −26.988 and the *p*-value was lower than 0.001. The distribution of the number of issued certificates for nurses and midwives was consistent with the normal distribution; the mean was 1557.42. After 2015, no increase was recorded, but rather a continuous decrease, below 500 certificates per year. In 2020, only 330 certificates were issued, but this may be related to the coronavirus pandemic and numerous travel restrictions.

Figure 2 presents the percentage distribution of the number of certificates issued from 2004–2020 for nurses and midwives. Certificates issued between 2004 and 2020 were stratified according to qualifications based on the National Chamber of Nurses and Midwives data. During the years 2004–2007, almost 50% of certificates were collected by graduates of medical secondary schools. This was followed by graduates of vocational schools —nurses. Over the years, the graduates of the above-mentioned faculties have requested fewer and fewer certificates. This was because young people are the ones who decide to go abroad and there are presently no medical high schools or medical vocational schools. A person who intends to work as a nurse or midwife must graduate from a bachelor’s or master’s degree of study in these fields. Currently, the most certificates are issued for graduates of bachelor’s degrees in nursing. This constitutes over 30% of all certificates issued for nurses and midwives. This trend started in 2010 and continues to this day. Furthermore, over the years, more and more graduates of master’s studies in nursing have received certificates; since 2011, this level has been at about 20%.

#### 3.1.3. The National Chamber of Physiotherapists Data

The National Chamber of Physiotherapists has issued certificates for their members since 2017 (legal establishment of this chamber was based on the Act on the profession of physiotherapists [30]). Until the end of 2020, it issued 1214 (Table 1) and then issued 50 in the first quarter of 2021. The distribution of the number of issued certificates was consistent with the normal distribution; the mean was 252.8, the standard deviation was 123.38. The coefficient of determination R^2^ was 0.6455.

#### 3.1.4. The Supreme Pharmaceutical Chamber and the Chamber of Laboratory Diagnosticians Data

According to the data obtained from the Supreme Pharmaceutical Chamber for the period of 2008–2020, the total number of certificates was 1460 (Table 1). The number of certificates collected by pharmacists in 2008 was the highest amounting to 173. In the following years there were irregular decreases and increases. However, the number of certificates did not exceed 150 per year. In the Figure 1c, three periods of decreases and increases in the number of certificates can be clearly distinguished. In the period of 2008–2011 the annual percentage change was −22.742, which was statistically significant, and the *p* value was equal to 0.025. The change between 2011 and 2015 was not statistically significant; the APC = 17.61, and *p*-value = 0.117. Another change observed in the period of 2015–2020 indicates a statistically significant decrease in the number of certificates issued, where the annual percentage change was −12.633 and the *p*-value was 0.023. Comparing 2019 to 2020, when countries faced the coronavirus pandemic, one cannot see a significant decline in the number of certificates—only 12 fewer were issued.

According to the data provided by the Office of the National Chamber of Laboratory Diagnosticians, from 11 March 2011 until mid-May 2021, only 158 certificates of professional qualifications needed to practice the profession of laboratory diagnostics in other EU Member States were issued.

### 3.2. The Main Destinations of Polish Medical Staff—Analysis of the European Database

The statistics published by the European Commission in the Regulated Profession Database [33] show the number of positive decisions taken on recognition of professional qualifications for the purpose of permanent establishment within the EU Member States and European Free Trading Association (EFTA) countries (Iceland, Liechtenstein, Norway, and Switzerland). The United Kingdom left the EU on 31 January 2020 and currently the Commission is in the process of updating some of the content on this platform following the withdrawal of the UK from the EU. However, since the UK is an important emigration destination for Polish health professionals, it is presented separately on the chart.

**Figure 3:**
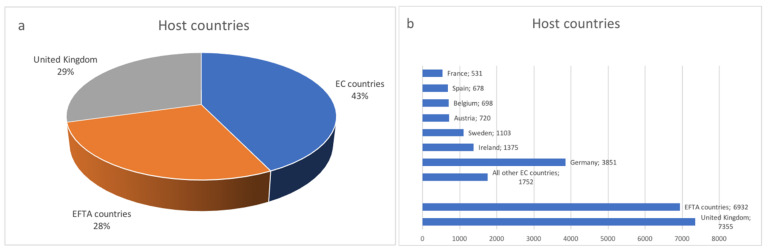
Host countries for medical staff qualified in Poland: (**a**) in total and (**b**) taking into account individual countries. Based on the European Commission. The EU Single Market. Regulated Profession Database (accessed on 4 April 2021), including Norwegian doctors who completed their studies in Poland.

According to data published in the Regulated Profession Database [33], since Poland’s accession to the EU, qualifications of 10,708 health professionals who acquired their diploma in Poland were recognised in other EU countries. 7355 health professionals found employment in the United Kingdom and 6930 in EFTA countries. Figure 3 presents information about countries in which migrating medical professionals had their qualifications recognised.

Approximately 3600 physicians who acquired their diploma in Poland have registered in another EU country and have had their professional qualifications formally recognized. The majority of these procedures have taken place in Germany (987), Sweden (663), Spain (597), and Ireland (585). Furthermore, over 6000 physicians have emigrated: to the United Kingdom (1825) and EFTA countries such as Norway (3970) and Switzerland (274).

It should be mentioned that Poland offers high quality medical study in English. Therefore, it is a very popular country for students from Norway who wish to study medicine. In the academic year 2017/18, about half of all Norwegian medical students were studying outside Norway (18% in Poland) [13]. According to the OECD Report, more than a half of foreign-trained physicians in Norway are in fact persons who were born in Norway and went abroad for medical study. The number of students from Norway studying medicine in Poland has increased significantly over the years. Since 2010 their number is estimated at around 1200.

Other medical professions are also quite willing to migrate to EC countries: nurses (3266), physiotherapists (1581), and dentists (756).

Detailed information concerning healthcare personnel trained in Poland whose diplomas were recognised in other European countries is presented in Table 3.

## 4. Discussion

**Figure 4:**
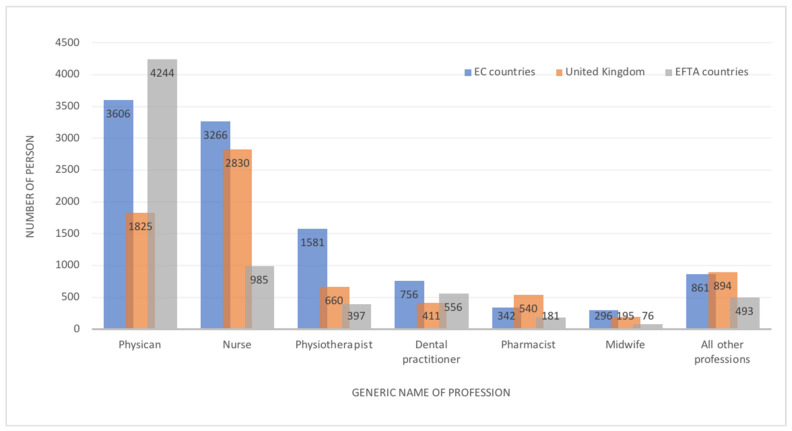
The most mobile medical professions (the total number from Poland’s accession to the EU to 2020), qualified in Poland who have recognised their diploma in the EC, UK, and EFTA countries. Based on the European Commission. The EU Single Market. Regulated Profession Database (accessed on 4 April 2021), including Norwegian doctors who completed their studies in Poland.

The results of our study show that the problem of Polish medical staff emigration is still under-researched. According to the data obtained from national chambers of health professionals, about 7–9% of practicing doctors and nurses have applied for certificates, which confirm their right to practice their profession in other Europan countries. The relatively high number of such certificates applied for by Polish physiotherapists (an average 300 per year) is also worrying, especially considering both the importance of this professional group for the system and the fact that relatively recently, the work of this professional group was legally regulated. Emigration among Polish pharmacists and laboratory diagnosticians is rather marginal. Moreover, information gathered from the European database confirmed that most professionals qualified in Poland who have decided to migrate are physicians, nurses, and physiotherapists (Figure 4). The countries most often chosen as emigration destinations were the United Kingdom, Germany, Sweden, Spain, Ireland, and EFTA countries (Norway and Switzerland).

The analysis of the data concerning emigration (emigration intentions) in Poland during the period in question shows some trends for the discussed individual professional groups and in the group as a whole. The largest number of certificates was issued in the years 2004–2007, i.e., right after Poland joined the EU. In 2008 after strong tensions and a period of protest, physicians obtained quite substantial salary raises [37]. This caused a significant drop in the number of physicians applying for competence certificates [38]. The number of doctors requesting the certificate fluctuated in the following years; but still, it dropped overall. A similar phenomenon can be noted for dentists and, to a certain extent, for pharmacists. Data for physiotherapists is limited (due to the more recent establishment of their professional chamber), but recent years also show a fall in the number of issued certificates. The data concerning nurses are slightly different. The above-mentioned physician protests caused some salary raises for nurses, which contributed to a falling number of these professionals interested in emigration. However, since the salary raises were rather limited, nurse dissatisfaction was growing. This led to both intensified protest actions by this professional group and their growing interest in emigration. This trend lasted till 2015 when they were promised (and to certain extent given) quit high salary raises. These raises were to be introduced consecutively over the coming 4 years. This caused a visible reduction of nurses applying for their qualification certificates (Table 1).

The reduced numbers of health professionals considering emigration or actually migrating can mostly be attributed to two factors: increased salaries these groups gained in the discussed period and the ageing of these groups. Research has confirmed that emigration intentions are negatively correlated with age and work experience: older and more experienced health professionals less often consider practicing medicine abroad [13,14,35,36,39].

Research from other countries also shows that younger medical professionals more often declared their intentions to migrate [39,40,41]. The current generation of young medical professionals value their work-life balance as a high priority; they expect not only higher earnings but also better working conditions and flexibility of working hours.

Job satisfaction is also negatively correlated with the intention to emigrate; those with higher levels of satisfaction are less interested in emigration [36,42]. The ones who stay seem to be more satisfied with their improved financial conditions. The reduced number of those who stay has decreased competition among them. This has forced healthcare facilities to pay health professionals more, as there are fewer of them and the demand for their work is much higher due to the ageing society’s needs.

Due to ageing societies and the ageing of the health workforce, the demand for doctors, nurses and other medical staff will continue to grow across European countries. International research has confirmed that health professional migration is particularly visible from Eastern and Southern Europe to richer European countries [43,44]. As a consequence, there will be a further outflow of medical staff from Poland and the current problems with staff shortages should continue due to still substantial differences in Southern/Eastern vs. Northern European wealth. Though the number of migrating health professionals has dropped substantially in the analysed period, it contributed to the staff shortages. Therefore, even a smaller number of migrants (compared to the period of 2004–2007) has significant impact on the Polish healthcare system.

The United Kingdom was for a long time one of the main destination countries for Polish migrants. As the number of migrants in recent years fell substantially overall, Brexit seems not to have affected the migration trend or to have affected it only slightly.

Other research confirmed that the vast majority of health professionals indicated a European country as their emigration destination. Among non-European destinations, the United States, Australia, and Canada are most often indicated [14,36].

To achieve sustainability, countries must focus their workforce policy and funding efforts on improving the retention of medical staff (including foreign-trained workers) and the performance of their workforce [45,46]. In many European countries, foreign-born doctors and nurses have contributed significantly to the growth in the total number of doctors and nurses in recent years. As was mentioned in the Introduction, the share of foreign-born professionals in Poland is insignificant. The share of foreign-trained doctors in Poland in 2017 was only 1.9%, much lower than the average of OECD countries (16%). This indicator for nurses is not even marginal, estimated at around 0.1% [13]. This is mainly due to the poor working conditions in the Polish healthcare system and the language barrier, but also the lack of a proactive recruitment policy.

As a response to significant staff shortages in Poland, in November 2020 the Act on amending certain acts in order to provide medical staff during the period when an epidemic emergency or epidemic is announced was enacted [47]. This new legal regulation was introduced to increase the size of the health workforce by simplifying the procedure for the employment of doctors, dentists, nurses, midwives, and paramedics with professional qualifications obtained outside the EU.

### 4.1. Implications of the Study and Recommendations for Further Research

Due to the significant staff shortages, the unfavourable age structure of health professionals, and the emigration of younger generations, Poland faces serious problems as a source country. The first implication from our study is to improve tools, mechanisms, and databases on the scale and reasons for medical staff emigration. Without reliable and comprehensive data, it is not possible to develop an effective monitoring procedure. Better cooperation and participation in international initiatives in this field are recommended in order to obtain more comprehensive and comparable data on migration. Moreover, improvement of national and regional policies and strategies on the health workforce is needed, based on a long-term, holistic approach instead of ad-hoc actions. There is evidence to suggest that policy-makers and healthcare managers need to consider combinations of different interventions (financial and non-financial) for the purpose of retaining health workers [48].

Due to the shortages in the most numerous professional groups (especially doctors and nurses), special attention should also be paid to other professions in the health labour market and their migration. All health professionals should be taken into account in the monitoring process, including those medical professions which are not listed in the EU Directive on the recognition of professional qualifications [1], but are important for the healthcare sector (see Table 3) and have also decided to emigrate. Paramedics are an example of such a profession and an interesting study on emigration intentions among Polish paramedics was conducted in 2017 [49]. The share of professionals in this group who declared the intention to migrate (11%) was significantly lower compared to nurses and doctors, but the excessive workload and long-term stress mentioned above (especially during the COVID-19 pandemic) could also increase the trend of emigration in this professional group.

Measurement and monitoring of medical staff migration is crucial in order for policy-makers and healthcare managers to implement proper actions and improve health workers retention. But it is equally important to understand the factors influencing migration decisions. If these motives and “push” factors are better known, appropriate strategies could be developed to prevent health professionals from leaving the Polish healthcare system. Attending knowledge about and identifying the factors influencing the decision to migrate should be one of the crucial issues of human resources management. In order to identify the nature of these factors and the predictors of emigration, further qualitative research in this field is recommended. It would be interesting to conduct qualitative research and explore “push” and “pull” factors among health professionals who have already started wok abroad and among those who have returned from emigration. Evidence based on qualitative and quantitative research will help to develop and implement a systemic approach to monitoring health professional migration.

### 4.2. Limitations of the Study

Our study is not free of limitations. Literature review confirms that Poland is still an under-researched country, especially in the field of health workforce policy. There are a few studies on emigration plans among medical students [50,51,52,53,54], but research among practicing professionals is scarce.

The next limitation is connected with the lack of a comprehensive database and reliable data on migration. It should be underlined that the available data on migration is fragmentary and incomplete, which is the main reason for the poor monitoring of health workforce migration. The trend of emigration is estimated based on the number of certificates issued by health professionals’ chambers. As mentioned in the article, these indicators only detail “intentions” to migrate, not the real scale of the phenomenon. Moreover, the years for which data from particular chambers are available, differ significantly, which makes it difficult to compare the emigration trends of the analysed health professionals. Some chambers provided information in the form of a cumulative number of issued certificates for the selected years. It is also likely that over the course of more than 15 years, there has been a change in the way the chambers collect data.

Data presented in the European database include information about foreign-trained doctors, nurses, and other professionals. Nevertheless, it should be highlighted that not all of the foreign-trained health professionals are foreigners, and that a large number in some countries (especially Norway and Sweden) are people born in the country who went to obtain a medical degree abroad before returning [13,33].

## 5. Conclusions

Currently, as a consequence of insufficient policies concerning the health workforce, the Polish system is facing serious problems; these include shortages, heavy workload and burnout, and the frustration and dissatisfaction of health personnel. Moreover, an inadequate level of remuneration and poor working conditions cause emigration among health professionals, and the further outflow of well-educated employees is a real challenge. Urgent implementation of a holistic approach and comprehensive health workforce policy is needed. Implementation of effective tools and mechanisms for monitoring emigration trends are necessary, but it is not possible to retain qualified health professionals without systemic improvement of working conditions within the Polish healthcare system (including increased salaries, reduced administrative burden, and facilitating working time).

## Figures and Tables

**Table 1:** Number of certificates confirming professional qualifications issued to health professionals from 2004–2020.

	Physicians	Dentists	Nurses and Midwives	Physiotherapists	Pharmacists	Total
2004–2009 *	7805	810	11,963	n/d	296	20,578 (3430 -annually)
2010	544	43	806	n/d	107	1500
2011	480	50	939	n/d	71	1540
2012	562	63	1154	n/d	103	1882
2013	441	55	1253	n/d	90	1839
2014	326	5	1324	n/d	147	1802
2015	249	5	1527	n/d	134	1915
2016	206	3	987	n/d	141	1337
2017	191	3	620	295	113	1222
2018	151	4	488	376	90	1109
2019	119	5	413	304	90	931
2020	79	3	330	239	78	729
Total	11,153	1049	21,804	1214	1460	36,680

* for pharmacists, data are available for the period 2008–2009.

**Table 2:** Results of the Joinpoint Regression Analysis—Annual Percentage Change (APC).

*Certificates Confirming Professional Qualification Issued to the Doctors*			
Period	APC	95% CI	*p* value
2010–2020	−17.605	(−20.225; −14.900)	<0.001
*Certificates confirming professional qualification issued to the dentists*			
Period	APC	95% CI	*p* value
2010–2012	36.664	(−55.981; 324.294)	0.445
2012–2015	−64.124	(−88.445; 11.381)	0.064
2015–2020	0.976	(−21.620; 30.088)	0.911
*All certificates issued to the doctors (including certificates of good standing)*			
Period	APC	95% CI	*p* value
2010–2015	−1.962	(−7.383; 3.777)	0.427
2015–2020	−10.517	(−15.465; −5.280)	0.003
*All certificates issued to the dentists (including certificates of good standing)*			
Period	APC	95% CI	*p* value
2010–2014	−0.468	(−4.857; 4.123)	0.807
2014–2015	−15.857	(−17.861; −13.804)	<0.001
*Certificates issued for the recognition of professional qualifications of pharmacist*			
Period	APC	95% CI	*p* value
2008–2011	−22.742	(−37.388; −4.671)	0.025
2011–2015	17.610	(−5.675; 46.645)	0.117
2015–2020	−12.633	(−21.511; −2.750)	0.023
*Certificates issued for the recognition of professional qualifications of nurses and midwives*			
2008–2010	−23.000	(−43.460; 4.864)	0.082
2010–2015	9.812	(−0.406; 21.079)	0.057
2015–2020	−26.988	(−31.860; −21.767)	<0.001

**Table 3:** Healthcare personnel qualified in Poland with qualifications recognised in other European countries.

Generic Name of Profession	Recognition in EC Countries	Recognition in the United Kingdom	Recognition in EFTA Countries	Recognition TOTAL
Doctor of medicine	3606	1825	4244 *	9675
Nurse	3266	2830	985	4534
Physiotherapist	1581	660	397	2638
Dental practitioner	756	411	556	1723
Pharmacist	342	540	181	1063
Midwife	296	195	76	567
Nursing assistant and health care assistant	208	2	198	408
Psychologist	155	78	82	315
Masseur/massage therapist/SPA therapist	82	0	0	82
Radiographer/radiotherapist	79	98	18	195
Medical/biomedical laboratory technician	75	122	24	221
Pharmaceutical technician/pharmaceutical assistant	57	52	59	168
Paramedic/ambulance nurse/other ambulance professionals	41	248	48	337
Dental technician	26	107	32	165
Dietician	24	19	2	45
Speech and language therapist	21	1	1	23
Optometrist (ophthalmic optician)	20	10	19	49
Geriatric nurse/carer for the aged	18	0	0	18
Chiropodist (podiatrist)	13	0	0	13
Dental hygienist	11	98	6	115
Bio-medical analyst	11	0	0	11
Dental assistant/dental nurse	5	52	0	57
Optician (dispensing optician)	4	1	0	5
Clinical psychologist	4	0	0	4
Hearing aid dispenser/audiometric technician/audiometric technician	2	5	3	10
Nutritionist/Clinical nutritionist	2	0	1	3
Nursery nurse	1	0	0	1
Surgical assistant	1	1	0	2
Psychomotor therapist	1	0	0	1
Total	10,708	7355	6932	22,448

Source: based on European Commission. The EU Single Market. Regulated Profession Database [33] * including Norwegian doctors who completed their studies in Poland.

## Data Availability

Not applicable.

## Supplementary Materials

The following supporting information can be downloaded at: https://www.mdpi.com/article/10.3390/ijerph19020940/s1, Table S1: Number of all certificates (confirming professional qualification and certificate of good standing) issued for doctors and dentists 2004–2020.
