# Supplementary material for: Exploration of Estimated Emigration Trends of Polish Health Professionals
Source: Int J Environ Res Public Health. 2022 Jan 14;19(2):940. doi: 10.3390/ijerph19020940 (PMC8776200; doi:10.3390/ijerph19020940)
Supplement: Supplementary file 1 [file ijerph-19-00940-s001.zip › ijerph-1474093-supplementary.pdf]

Supplementary Material

Table S1. Number of all certificates (confirming professional qualification and certificates of good standing) issued for doctors and dentists 2004-2020

Year	Number of all certificates issued for doctors	Including graduates of the Medical State Exam	Number of all certificates issued for dentist	Including graduates of the Medical-Dental State Exam	Total number of certificates issued in selected years
2004-2009	16 032	897	4 263	34	21 192
2010	1 251	194	357	40	1 802
2011	1 094	209	351	46	1 654
2012	1 237	283	342	54	1 862
2013	1 110	173	338	44	1 621
2014	1 151	25	378	5	1 554
2015	1 087	17	284	4	1 388
2016	901	28	247	3	1 176
2017	939	27	186	3	1 152
2018	805	16	174	3	995
2019	741	11	145	5	897
2020	588	10	129	3	727
**Total**	**26 936**	**1 890**	**7 242**	**244**	**36 263**

Source: data from the National Chamber of Physicians

The data in Table S1 presents information on the certificates issued for doctors and dentists during the years 2004-2020. Persons who had been issued at least one type of certificate are included, both certificates confirming their qualifications and certificates of good standing (valid for only 3 months).
